# Sequence Comparisons of Odorant Receptors among Tortricid Moths Reveal Different Rates of Molecular Evolution among Family Members

**DOI:** 10.1371/journal.pone.0038391

**Published:** 2012-06-11

**Authors:** Colm Carraher, Astrid Authier, Bernd Steinwender, Richard D. Newcomb

**Affiliations:** 1 The New Zealand Institute for Plant & Food Research Limited, Auckland, New Zealand; 2 School of Biological Sciences, University of Auckland, Auckland, New Zealand; 3 The Allan Wilson Centre for Molecular Ecology and Evolution, Auckland, New Zealand; New Mexico State University, United States of America

## Abstract

In insects, odorant receptors detect volatile cues involved in behaviours such as mate recognition, food location and oviposition. We have investigated the evolution of three odorant receptors from five species within the moth genera *Ctenopseustis* and *Planotrotrix,* family Tortricidae, which fall into distinct clades within the odorant receptor multigene family. One receptor is the orthologue of the co-receptor Or83b, now known as Orco (OR2), and encodes the obligate ion channel subunit of the receptor complex. In comparison, the other two receptors, OR1 and OR3, are ligand-binding receptor subunits, activated by volatile compounds produced by plants - methyl salicylate and citral, respectively. Rates of sequence evolution at non-synonymous sites were significantly higher in OR1 compared with OR2 and OR3. Within the dataset OR1 contains 109 variable amino acid positions that are distributed evenly across the entire protein including transmembrane helices, loop regions and termini, while OR2 and OR3 contain 18 and 16 variable sites, respectively. OR2 shows a high level of amino acid conservation as expected due to its essential role in odour detection; however we found unexpected differences in the rate of evolution between two ligand-binding odorant receptors, OR1 and OR3. OR3 shows high sequence conservation suggestive of a conserved role in odour reception, whereas the higher rate of evolution observed in OR1, particularly at non-synonymous sites, may be suggestive of relaxed constraint, perhaps associated with the loss of an ancestral role in sex pheromone reception.

## Introduction

The sensing of volatile compounds or olfaction is essential for insects that use chemical cues in such behaviours as mate recognition, food location and oviposition. To perceive odours insects use a novel family of receptors. While mammalian odorant receptors are classical G protein-coupled receptors, recent evidence shows that insect odorant receptors (ORs) act predominantly as ligand-gated cation channels [1], [2], [3]. However, there is also evidence suggesting that insect ORs may be able to signal via classical G protein pathways [2]. To address these data Nakagawa and Vosshall [4] have proposed a consensus model that supports a dual mechanism where the insect ORs operate via both ionotropic and metabotropic pathways. Like mammalian odorant receptors, insect ORs also contain seven transmembrane regions, however they are orientated in the opposite orientation in the plasma membrane, with their N terminus instead located in the cytoplasm [3], [5], [6]. One highly conserved member of the insect OR family, Or83b, is essential for olfactory ability [5], [7]. When this receptor is mutated in Drosophila the flies are anosmic, but this mutation can be rescued by replacement, even with orthologues from other insect orders. Or83b is required to form the ion channel, partnering with the other ligand-binding members of the family to produce a functional heteromeric odorant-sensitive receptor complex, although the details of the structure and mechanism of the complex remain scant. Recently, Or83b has been renamed Orco, short for odorant receptor co-receptor [8]. Many of the ligand-binding ORs from Drosophila have been deorphaned revealing that these ligand-binding receptors are broadly tuned, predominantly to compounds associated with fruit such as esters, alcohols and aldehydes [9], [10].

**Figure 1:**
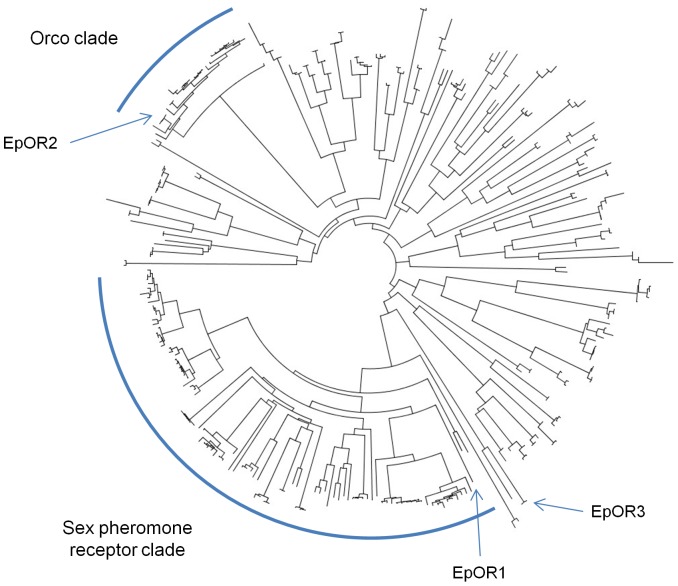
Unrooted phylogenetic tree of all lepidopteran odorant receptors within Genbank as of 8 November, 2010. The neighbour joining tree was constructed from dayhoff amino acid distances. The positions of OR1, OR2 (Orco), and OR3 from *Epiphyas postvittana* are indicated with arrows (EpOR1, EpOR2, and EpOR3), while the Orco and sex pheromone receptor clades are highlighted by semicircles.

Comparative studies of ORs across the Drosophila genus have provided some insights into the evolution of this large multigene family. The birth and death model of gene family evolution seems to fit well the broad patterns of evolution of the family across the genus [11], [12], [13], [14]. There are many cases of gene gain through duplication, as well as gene loss. The Orco subunit shows ω values consistent with being under strong purifying selection [14], where as some ligand-binding ORs show evidence of being under positive selection, particularly those associated with detecting specific fruit esters [15], [16], [17]. While amino acid variation and putatively selected sites are equally distributed across different structural regions including the N and C termini, internal and external loops and transmembrane regions [18], the C terminal regions of the receptors are more highly conserved compared with the N terminal regions [17]. To date however, little research has addressed whether these patterns of variation and multigene family evolution observed in Drosophila ORs extrapolate to ORs from other insects orders.

Genes encoding odorant receptors are being isolated from an increasing number of species within the insect order Lepidoptera. As well as orthologues of Orco, other classes of receptors are emerging, including receptors involved in detecting sex pheromones and receptors tuned to particular classes of plant volatiles. Receptors involved in the detection of sex pheromone produced by female moths have been isolated from a number of species of Lepidoptera, mainly within the families Bombycidae and Noctuidae [19], [20], [21], [22], [23]. These receptors are typically male-biased in their expression and fall into a distinct phylogenetic clade. Odorant receptors that are female-biased in their expression have also been identified and characterised in *Bombyx mori* [24], with receptors characterised for their ability to bind compounds such a linalool, benzoic acid, 2-phenyl ethanol and benzaldehyde that were suggested to be part of a yet to be described male pheromone [25]. Other groups of conserved ORs have been identified within the Lepidoptera. One OR that is conserved across many families of Lepidoptera involved in detecting citral [26], a second group confined to the Noctuidae [27], and others identified through a comparison of odorant receptors from *B. mori*, *Heliothis virescens* and *Manduca sexta* [28]. Besides these, a receptor expressed in the larvae of *B. mori* has been described (BmOR56) that binds the plant volatile cis-jasmone [29].

Three odorant receptors have been isolated from the tortricid pest, *Epiphyas postvittana* [26]. These three receptors each fall into different major clades within the odorant receptor multigene family from moths (Figure 1) and have different roles in olfaction. One odorant receptor, EpOR2, is an orthologue of Orco, while EpOR1 and EpOR3 are activated by plant volatiles. EpOR1 recognises a range of compounds including plant terpenoids and the compound methyl salicylate. Of the compounds tested EpOR1 is best activated by methyl salicylate, which is an important plant semiochemical that alerts other plants of impending pests and pathogens. Phylogenetically, EpOR1 falls inside the sex pheromone receptor clade, however this receptor does not bind components of the *E. postvittana* sex pheromone, nor does it show male-biased expression [26]. EpOR3 best binds the monoterpenes citral, a racemic mixture of the isomers geraniol and nerol. Orthologues of this receptor have been identified across a number of lepidopteran families with the orthologue from the silkworm, *Bombyx mori* (BmOR49), also able to bind citral.

Species members of two New Zealand endemic genera of leafroller moths *Ctenopseustis* and *Planotortrix* (*C. obliquana, C. herana, P. octo, P. excessana, P. notophaea*), like *E. postvittana*, are polyphagus pests of horticulture and forestry. There has been significant interest in the chemistry, biosynthesis and evolution of the sex pheromones used by these species [30], [31]. Many of the sex pheromone blends contain uncommon pheromone components such as (*Z*)-5-tetradecenyl acetate, (*Z*)-7-tetradecenyl acetate and (*Z*)-8-tetradecenyl acetate that are produced by a variety of different enzyme activities including Δ5, Δ9, and Δ10 desaturation. For example *C. obliquana* uses a blend of (*Z*)-5-tetradecenyl acetate and (*Z*)-8-tetradecenyl acetate as its sex pheromone, where as *C. herana* has lost the expression of the Δ10 desaturase which produces the (*Z*)-8-tetradecenyl acetate to result in the production of a pure (*Z*)-5-tetradecenyl acetate sex pheromone for this species [30]. Similarly in the *Planotortrix* genus the gain of expression of a Δ10 desaturase is involved in the evolution of the *P. octo* sex pheromone, which is predominantly composed of (*Z*)-8-tetradecenyl acetate, whereas the related species *P. excessana* uses a blend of (*Z*)-5-tetradecenyl acetate and (*Z*)-7-tetradecenyl acetate [30]. The speciation events that have given rise to many of the species in these two genera look to have arisen relatively recently, with molecular clock estimates suggesting a time to common ancestor for *C. obliquana* and *C. herana* as well as *P. octo* and *P. excessana* of 500,000 years ago [32].

Here we report the isolation of orthologues of EpOR1, EpOR2 and EpOR3 from *Ctenopseustis obliquana*, *C. herana*, *Planotortrix octo, P. excessana* and *P. notophaea*. Analyses of their sequences reveal major differences in rates of evolution among the receptors, with OR1 displaying higher substitution rates compared with the other two receptors, while OR3 appears to be a Lepidoptera-specific ligand-binding OR that is as conserved as Orco.

## Materials and Methods

### Moth Colonies, Moth Rearing, DNA and RNA Preparation

Native leafroller moths from each of the five species *Planotortrix octo, P. excessana, P. notophaea, Ctenopseustis herana,* and *C. obliquana*, are from laboratory colonies held at the New Zealand Institute for Plant & Food Research in Auckland, New Zealand. Collection details of these colonies are as described in Newcomb and Gleeson [31].

Antennae were removed from 100 male moths of each species and immediately snap frozen in liquid nitrogen. Total RNA was extracted using TRIzol reagent (Invitrogen, Carlsbad, CA, USA) as per the manufacturer’s instructions, and treated with DNAseI Amplification Grade (Invitrogen). Reverse transcription was carried out using SuperScript III as per the manufacturer’s instructions (Invitrogen). One µg of total RNA was reverse transcribed using an Oligo dT18 primer (Invitrogen) and random hexamers (Promega). cDNA synthesis was carried out at 50°C for 1 hour, followed by 70°C for 15 minutes. The resulting cDNA was diluted 1∶3 with water prior to use in PCRs.

**Table 1:** Summary statistics for odorant receptors OR1, OR2 and OR3 from *Ctenopseustis* and *Planotortrix* species.

Receptor	s^a^	N^b^	S^c^	k^d^	dN/dS M0^e^	dN/dS M3^f^
OR1	5	392	0.58	2.30	0.61	0.61
OR2	5	472	0.45	3.11	0.04	0.04
OR3	5	410	0.28	2.61	0.07	0.08

a number of sequences.

b number of codons.

c tree length.

d transition/transversion ratio.

e dN/dS under M0.

f dN/dS under M3.

### PCR, Cloning and Sequencing

Initial PCR primers for each of the three odorant receptors were designed from existing *Epiphyas postvittana* sequences (EU791886.1, EU791887.1, EU791888.1; Jordan et al 2009). These primers were then modified after isolating and sequencing the 5′ and 3′end of the cds by RACE. The final primers used to amplify full or near full length copies of the cDNAs were OR1 Full Forward (5′-ATGGAGGTATTTGATTTGGGATAC-3′), OR1 Full Reverse (5′-TTARTTGGCAATGTATTCAGCATCAT-3′), EposOR2expresssf9 (5′-CTCGAGATGATGGGGAAGGTGAAA-3′), natOR2R5 (5′-TTGCACCAACACCATGAAGT-3′), 5′OR3F1 (5′-ATGGAAGAGACCATCCGAACCTTC-3′) and 3′OR3R1 (5′-GTTTTCATCAAACACTGACATCACC-3′). Standard PCR amplifications were carried out in 50 µL reaction volumes containing 0.5 U Platinum *Taq* polymerase (Invitrogen), 1× reaction buffer, 1.25 mM magnesium chloride, 0.2 mM dNTP mix, and 0.2 µM of each primer, with 1 µL of 1∶3 diluted cDNA. PCR amplifications were performed on a GeneAmp 9700 (Applied Biosystems) PCR machine with an initial denaturation step of 2 min at 94°C, followed by 35 cycles (94°C for 30 s, 55°C for 30 s, 72°C for 1–1.5 min), and then a final elongation step at 72°C for 10 min. PCR products were cloned into the pGEM-T-easy vector (Promega) and clones sequenced for each OR from each species until at least two identical sequences were recovered from independent clones. Sanger sequencing was carried out at the Allan Wilson Centre Genome Service at Massey University, Palmerston North, New Zealand, using M13 forward and reverse primers.

**Table 2:** Amino acid identity matrix for *Ctenopseustis* and *Planotortrix* orthologues of OR1, OR2 and OR2.

		Cher^a^		Cobl^b^		Pexc^c^		Pnot^d^		Poct^e^		Epos^f^	
OR1													
Cobl	95.4												
Pexc	80.5		79.7										
Pnot	77.7		77.0		88.1								
Poct	80.3		79.5		98.7		87.6						
Epos	60.5		60.0		61.3		59.0		61.0				
Bmor^g^	32.6		32.9		33.3		34.0		33.1		33.9		
OR2													
Cobl		99.6											
Pexc		97.7		97.7									
Pnot		96.8		96.8		98.7							
Poct		97.5		97.5		99.8		98.5					
Epos		95.1		95.1		96.4		96.4		96.2			
Bmor^h^		83.5		83.3		84.0		83.3		84.0		84.4	
OR3													
Cher		99.3											
Pexc		97.1		96.8									
Pnot		97.1		97.1		98.3							
Poct		97.1		96.8		100		98.3					
Epos		89.5		89.3		89.0		88.8		89.0			
Bmor^i^		65.4		65.1		65.1		65.1		65.1		65.4	

a Ctenopseustis obliquana.

b C. herana.

c Planotortrix excessana.

d P. octo.

e P. notophaea.

f Epiphyas postvittana.

g BmOR1.

h BmOR2.

i BmOR49.

**Figure 2:**
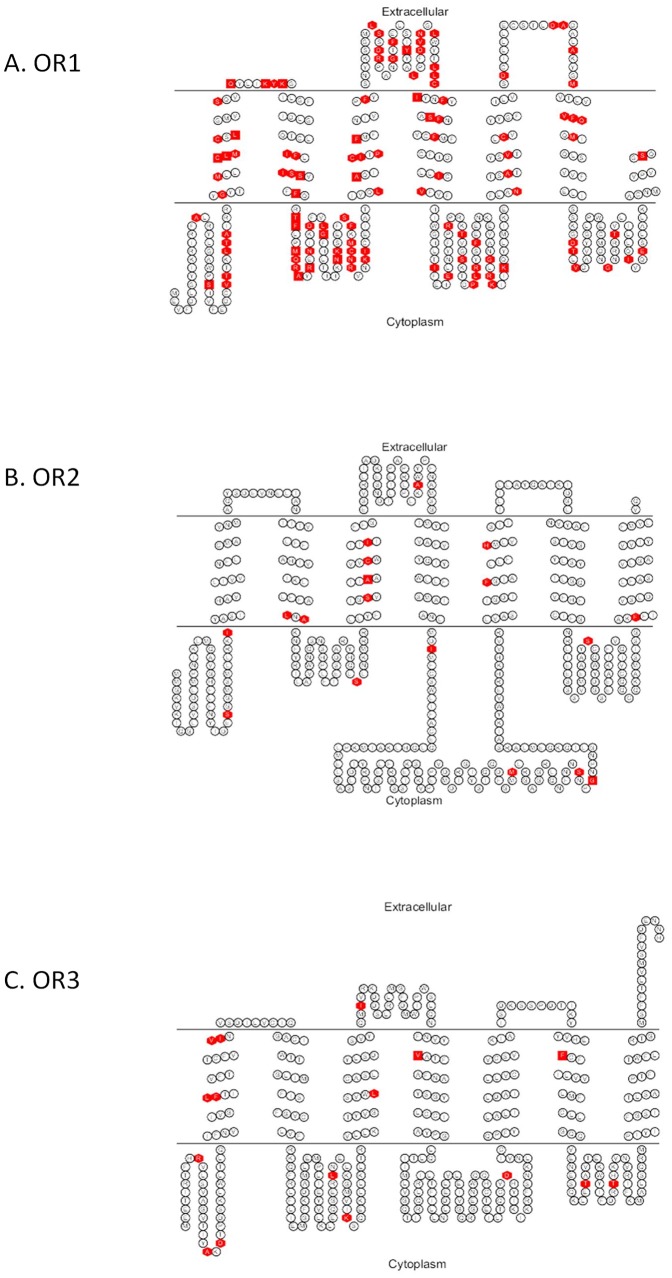
Predicted transmembrane topologies of OR1 (A), OR2 (B) and OR3 (C), with variable sites highlighted in red. The double line indicates the membrane region, with extracellular and cytoplasmic sides labelled.

**Figure 3:**
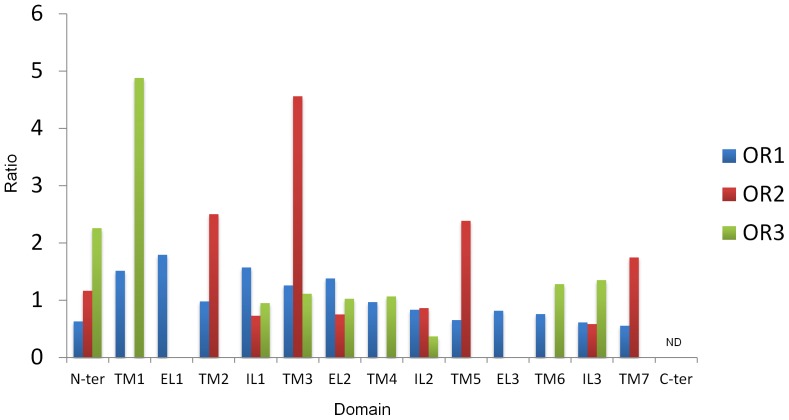
Ratio of the relative amino acid differences per domain averaged for OR1, OR2 (Orco) and OR3 across *Ctenopseustis obliquana*, *C. herana*, *Planotortrix octo*, *P. excessana* and *P. notophaea*. The ratio for each domain is the average of the number of amino acid differences divided by the number of expected differences. Expected differences were calculated by multiplying the length of the domain by the total number of differences per protein then dividing by the length of the protein. The ratio would be 1 if the amino acid changes occurred at the same rate across the entire protein. N-ter = N terminus; TM1-TM7 = transmembrane domains 1–7; IL1-3 = internal loops 1–3; EL1-3 = external loops 1–3; C-ter = C terminus. ND = not determined.

**Figure 4:**
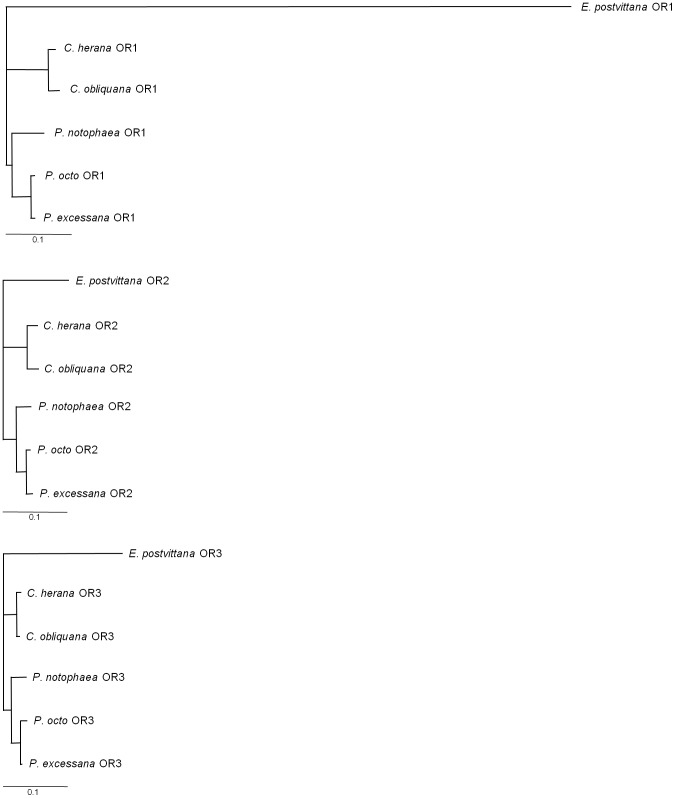
Phylogenetic trees of ortholgues of OR1, OR2 (Orco) and OR3 from the leafroller species *Ctenopseustis obliquana*, *C. herana*, *Planotortrix octo*, *P. excessana* and *P. notophaea*, with *Epiphyas postvittana* as an outgroup. Maximum likelihood trees were constructed from nucleotide sequences of the coding regions of each receptor using the HKY85 model based on ModelTest output.

**Table 3:** Likelihood ratio tests between concatenated sequences.

	Model A Lnl	Model B Lnl	2×DLnl	*P* value
OR1+OR2+OR3 concatenated	7802.20	7825.37	46.34	8.64E-11
OR1+OR2 concatenated	5560.01	5553.69	12.64	3.77E-04
OR1+OR3 concatenated	4993.92	4970.91	46.02	1.17E-11
OR2+OR3 concatenated	5029.84	5023.16	13.36	2.57E-04

model A assumes complete homogeneity among genes, while model B assumes different substitution rates but the same pattern of nucleotide substitution for each gene.

## Discussion

### Sequence Analysis

Sequence data was edited manually using Sequencher (Gene Codes) and amino acid and nucleotide sequences were aligned using ClustalX with the *Epiphyas postvittana* sequences. The start codon for each gene was taken as the methionine that aligned with the first methionine in the *E. postvittana* sequences. Gaps in the coding sequence alignment were removed for the PAML analysis. Maximum likelihood trees were generated using the PHYML [33] plugin in Geneious [34], with a model chosen by ModelTest [35].The dN and dS rates were estimated using the codon-based substitution models in PAML version 4.4c [36], using the model M0, which has one ω ratio for all sites, and M3 [37], which has three categories of site with the ω ratio free to vary for each site class. Evidence for positively selected sites was tested using the M8 model in PAML. The “beta plus ω” selection model, has eight categories of site from a beta distribution, plus an additional category of site that has a ω ratio free to vary from 0 to >1. These models are described in detail in [37], [38], [39], [40].

Tests for differences in substitution rates among genes were conducted using two methods. First, we compared ranks of non-synonymous rates (M3) by branch for pairs of receptor genes using a Mann-Whitney U test. Second, using the likelihood ratio test, we compared a fixed model of dN/dS derived from concatenated sequences of receptors (model A) with a model where the rate of a partitioned receptor gene was allowed to vary (model B). This approach allowed us to test for differences in rates between the genes within each species. If there is a difference between the two rate estimates then it can be asserted that the genes are evolving at different rates [41].

Consensus transmembrane domains were predicted using TMHMM [42] at the transmembrane prediction server (http://www.cbs.dtu.dk/services/TMHMM/), and TMPred [43] at the server (http://www.ch.embnet.org/software/TMPRED_form.html). The predicted domains from both were very similar. Where they disagreed an averaged consensus was produced. The topology diagrams were constructed using TOPO2 Transmembrane Protein Display [44] by the server at (http://www.sacs.ucsf.edu/TOPO-run/wtopo.pl).

## Results

Orthologues of three odorant receptor genes, OR1, OR2 and OR3 were isolated and sequenced from antennal cDNA of five leafroller species; *Ctenopseustis obliquana*, *C. herana*, *Planotortrix excessana*, *P. octo* and *P. notophaea*. All the coding regions of the three receptors except for the C-terminal 19 codons of OR1 and the terminal codon of OR2 were sequenced from orthologues of all five species. Unfortunately for OR1 and OR2 we could not identify primers further 3′ that could be used to consistently amplify cDNAs of these genes from all five species. Sequences are available in Genbank under accession numbers HQ619206-HQ619220. Alignments of the predicted ORs for each of the five orthologues for the three receptors, together with EpOR1, EpOR2 and EpOR3 of the related tortricid, *Epiphyas postvittana* [26] are presented in Figures S1, S2, and S3. There is good bootstrap support (1000 bootstrap replicates) for the monophyly of the native leafroller species with *E. postvittana* are 94.4%, 91.8% and 96.0% for OR1, OR2 and OR3, respectively (Figure 1). The summary statistics for each set of receptors from the five species are presented in Table 1. dN/dS values among the three OR genes under the M3 model range from 0.04 for OR2, to 0.08 for OR3 to 0.61 for OR1. Within each gene, however, likelihood ratios tests failed to find any significant (P>0.05) evidence for positive selection in M0 vs M3 (2Δl = 0; 4.75; 4.64), M7 vs M8 (2Δl = 1.05; 2.01; 2.22) or M8a vs M8 (2Δl = 0.004; 0; 0) comparisons for OR1, OR2 or OR3, respectively.

Amino acid identities across the orthologues of OR 1, 2 and 3 from the *Ctenopseustis* and *Planotortrix* species, *E. postvittana* and putative orthologues from the silkworm *Bombyx mori* (BmOR1, BmOR2 and BmOR49, respectively) were calculated and are presented in Table 2. Of the three receptors, OR2 and OR3 have the highest levels of amino acid identities among the species, with within genera comparisons (*Ctenopseustis* and *Planotortrix*) ranging from 98.3% to 100% for OR3 and OR2, while OR1 is the least conserved, with within genera comparisons ranging from 87.6% to 98.7%. Between genera comparisons range from as high as 97.7% for OR2 to as little as 77.0% for OR1. Amino acid identities with *E. postvittana* orthologues are lower again, as low as 59.0% for OR1, 88.8% for OR3, and 95.1% for OR2. Comparisons with receptors from *B. mori* result in much lower amino acid identities, down to 32.6% to 34.0% for OR1, 65.1% to 65.4% for OR3 and 83.3% to 84.4% for OR2.

We then examined how the amino acid sequence variation was distributed within the receptors. Overall within our dataset, OR1 contains 109 variable sites, while OR2 and OR3 contain 18 and 16 variable sites, respectively (Figure 2). Transmembrane topologies were predicted for each receptor from a consensus of two independent prediction algorithms to examine where the variation was located within each receptor. Based on the topology, each OR could be broken into fifteen regions, including the intracellular N terminal region, the seven transmembrane regions, the three intracellular loops, the three extracellular loops, and the C terminal region. We first examined how amino acid variation within each of the three odorant receptors was distributed graphically by plotting the ratio of the relative number of amino acid differences for each region (Figure 3). From this graphical analysis the relative levels of variation are high in the transmembrane regions of OR2 in TM2, TM3, TM5 and TM7, while OR3 shows high levels of variation only in TM1. Variation in OR1 looks equally distributed across all fifteen regions. Statistically, no differences in the frequency of variable sites could be detected across the fifteen regions within each of the three receptors using χ^2^ tests (OR1: χ^2^ = 15.21 *P* = 0.29, OR2: χ^2^ = 18.38 *P* = 0.14, OR3: χ^2^ = 16.26 *P* = 0.30).

Finally, we examined variation and rates of evolution among the three odorant receptor genes. Phylogenetic analyses for each receptor are presented in Figure 4. The three gene trees show the same pattern of relationships among the species. As described above dN/dS values among the three OR genes under the M3 model range from 0.04 for OR2, to 0.08 for OR3 to 0.61 for OR1. Non-synonymous (dN) and synonymous (dS) substitution rates are given for each branch in the tree for the three receptors in Figure S4. The rates of non-synonymous substitution in OR1 are consistently higher at every branch in the phylogeny than for OR2 (*U* = 24, *P* = 0.008) and OR3 (*U* = 23, *P* = 0.014), with no difference in non-synonymous substitution rate between OR2 and OR3 (*U* = 13, *P* = 0.458) using the non-parametric Mann-Whitney U test. There are no significant differences among the rates of synonymous substitutions for the three genes (data not shown). Further evidence for rate differences between the genes was generated using PAML by comparing dN/dS from a model where substitution rates are fixed across all genes and a model where they are allowed to vary within each partitioned gene within a concatenated dataset. Using likelihood ratio tests all gene to gene comparisons showed significant evidence for rate differences among the genes (Table 3).

We have sequenced orthologues of three odorant receptors across two genera (*Ctenopseustis* and *Planotortrix*) of tortricid moths from distinct phylogenetic positions within the odorant receptor multigene family of the Lepidoptera. The three odorant receptors (OR1, OR2 and OR3) are not evolving at similar rates. Among the leafroller species the levels of amino acid identity are highest and the rates of non-synonymous substitution the lowest for Orco, OR2. Orco is essential for olfaction, performing central roles in ligand-binding subunit trafficking and ion channel function in the receptor complex [5]. Consistent with these essential roles, dN/dS values for the moth orthologues are considerably less than 1 (dN/dS[M3] = 0.04). These results suggest that as has been found for Orco across the Drosophila genus [14], this gene within the Lepidoptera is also under strong purifying selection.

Substitution rates and dN/dS values across the leafroller moths are also low for the receptor OR3 (dN/dS[M3] = 0.08) and this receptor shows high levels of sequence conservation particularly at the C terminal end of the protein across the Lepidoptera [26]. This is perhaps more surprising as this receptor is involved in ligand-binding with no evidence that it plays any essential structural or functional roles in receptor complexes. OR3 in *E. postvittana* best binds the monoterpene isomers neral and geranial that make up the racemic mixture known as citral, and furthermore the distant orthologue of OR3 from *Bombyx mori*, OR49, also binds citral. Since OR3 is highly conserved across the Lepidoptera we can speculate that its role in binding citral will also be conserved. In *E. postvittana* citral is an oviposition deterrent [45]; however whether citral has this role in other moth species is still to be determined. No orthologues of OR3 have been found in any other insect orders suggesting this role is lepidopteran-specific.

OR1 across the leafroller moths is evolving at a much faster rate than OR2 and OR3, especially in terms of non-synonymous substitutions (dN/dS[M3] = 0.61). This high level of variation may be suggestive of a relaxation of the constraint on purifying selection on OR1 compared with the other two receptors. Further evidence that this increased level of variation is from relaxed constraint, rather than say positive selection, comes from the observation that the variation is distributed evenly right across the receptor and not in any particular region, for example those more likely involved in ligand binding, such as transmembrane regions and extracellular loops. In *E. postvittana* OR1 detects a range of plant volatiles, but best detects the plant defence compound, methyl salicylate [26]. The phylogenetic position of OR1 within the pheromone receptor clade may be suggestive of a scenario where this receptor was once able to detect sex pheromone components, but has now become freed from this function. Certainly OR1 seems to be confined to within the tortricidae with the only other orthologue identified to date in the closely related tortricid, *E. postvittana*, suggesting that these evolutionary scenarios are highly specific to this family of moths.

Another possible explanation for the high rates of non-synonymous variation in OR1 is that the gene is duplicated within some or all of these species and that the perceived high rates are merely a consequence of comparing paralogous rather than strictly orthologous genes. We checked this scenario by examining preliminary assemblies of the *C. obliquana* and *P. octo* genomes and conducting both quantitative and normal PCR experiments on genomic DNA. While we did identify further relatives of OR1 from the pheromone receptor clade in these species, we could not detect more than one copy of OR1 (or OR1 and OR3) using quantitative PCR from genomic DNA in *C. obliquana*, *C. herana, P. octo* and *P. excessana* (Figure S5), giving us confidence we are comparing true orthologues.

Therefore as has been described for odorant receptors across the Drosophila genus [14], [15], [16], we have found considerable heterogeneity in the rates of evolution across different odorant receptors in two sibling genera within the Lepidoptera. This variation ranges from sequence conservation in orthologues of the essential subunit Orco and a receptor that responds to the plant volatile citral, to evidence for high rates of non-synonymous evolution in an odorant receptor that falls with the lepidopteran-specific sex pheromone receptor clade.

## Supporting Information

Figure S1 **Amino acid alignment of OR1 from** ***Planotortrix octo, P. excessana, P. notophaea Ctenopseustis obliquana, C. herana and Epiphyas postvittana.***
(PDF)

Figure S2 **Amino acid alignment of OR2 from** ***Planotortrix octo, P. excessana, P. notophaea Ctenopseustis obliquana, C. herana and Epiphyas postvittana.***
(PDF)

Figure S3 **Amino acid alignment of OR3 from** ***Planotortrix octo, P. excessana, P. notophaea Ctenopseustis obliquana, C. herana and Epiphyas postvittana.***
(PDF)

Figure S4 **Non-synonymous and synonymous rates for all branches of OR1, OR2 and OR3 trees of** ***Ctenopseustis obliquana*** **,** ***C. herana*** **,** ***Planotortrix octo*** **,** ***P. excessana*** **and** ***P. notophaea*** **, with** ***Epiphyas postvittana*** **as an outgroup, plotted onto a consensus tree.** Rates were calculated based on the M3 model in PAML, which has three categories of site with ω free to vary for each site category.
(PDF)

Figure S5 **Ratio of OR1, OR2 and OR3 to α-tubulin and EF-1α from genomic DNA.**
(PDF)
