# Supplementary figures and images for: Sequence Comparisons of Odorant Receptors among Tortricid Moths Reveal Different Rates of Molecular Evolution among Family Members
Source: PLoS One. 2012 Jun 11;7(6):e38391. doi: 10.1371/journal.pone.0038391 (PMC3372514; doi:10.1371/journal.pone.0038391)

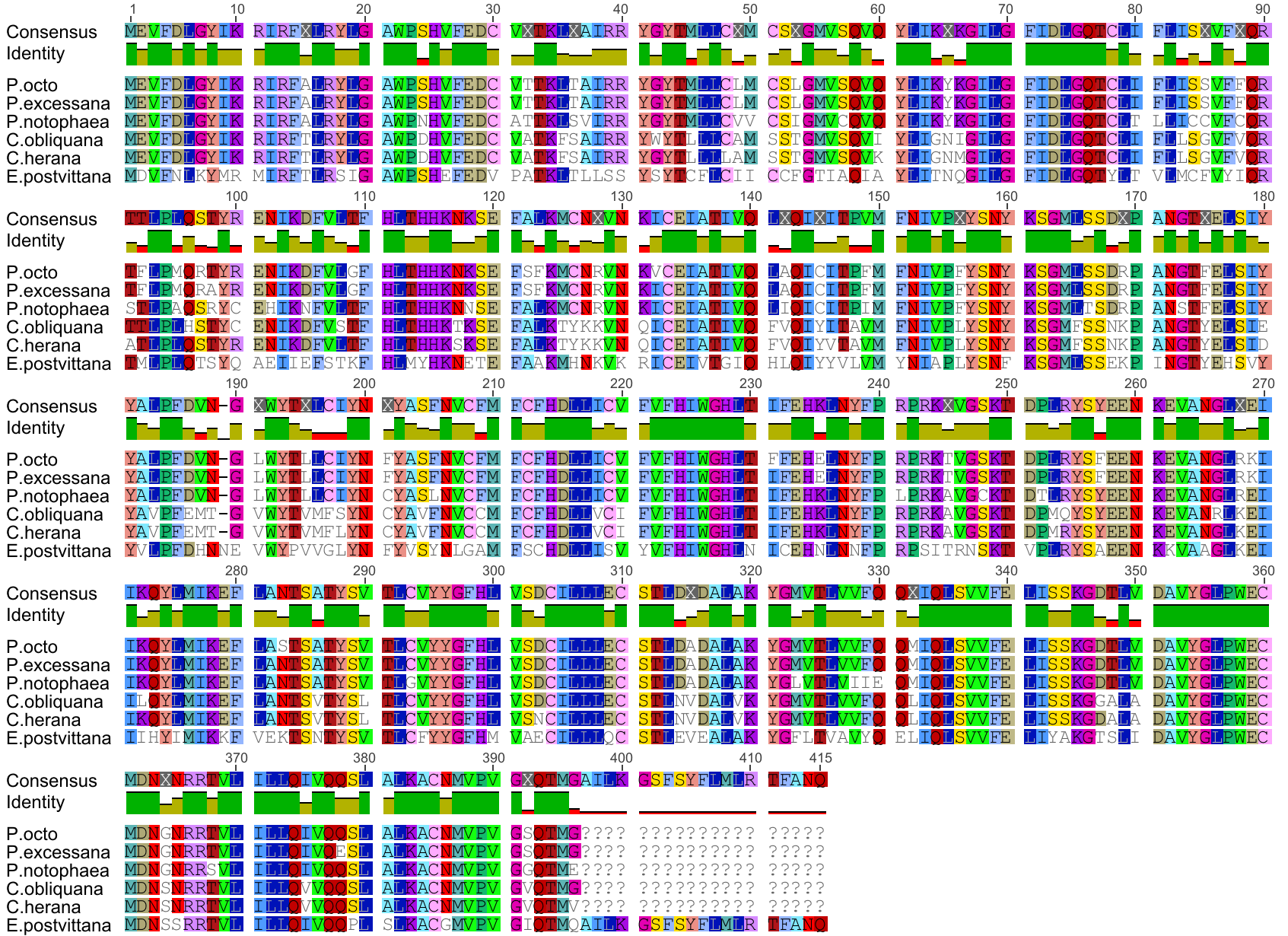

Supplement: Figure S1 — Amino acid alignment of OR1 from Planotortrix octo, P. excessana, P. notophaea Ctenopseustis obliquana, C. herana and Epiphyas postvittana. (PDF) [file pone.0038391.s001.pdf]

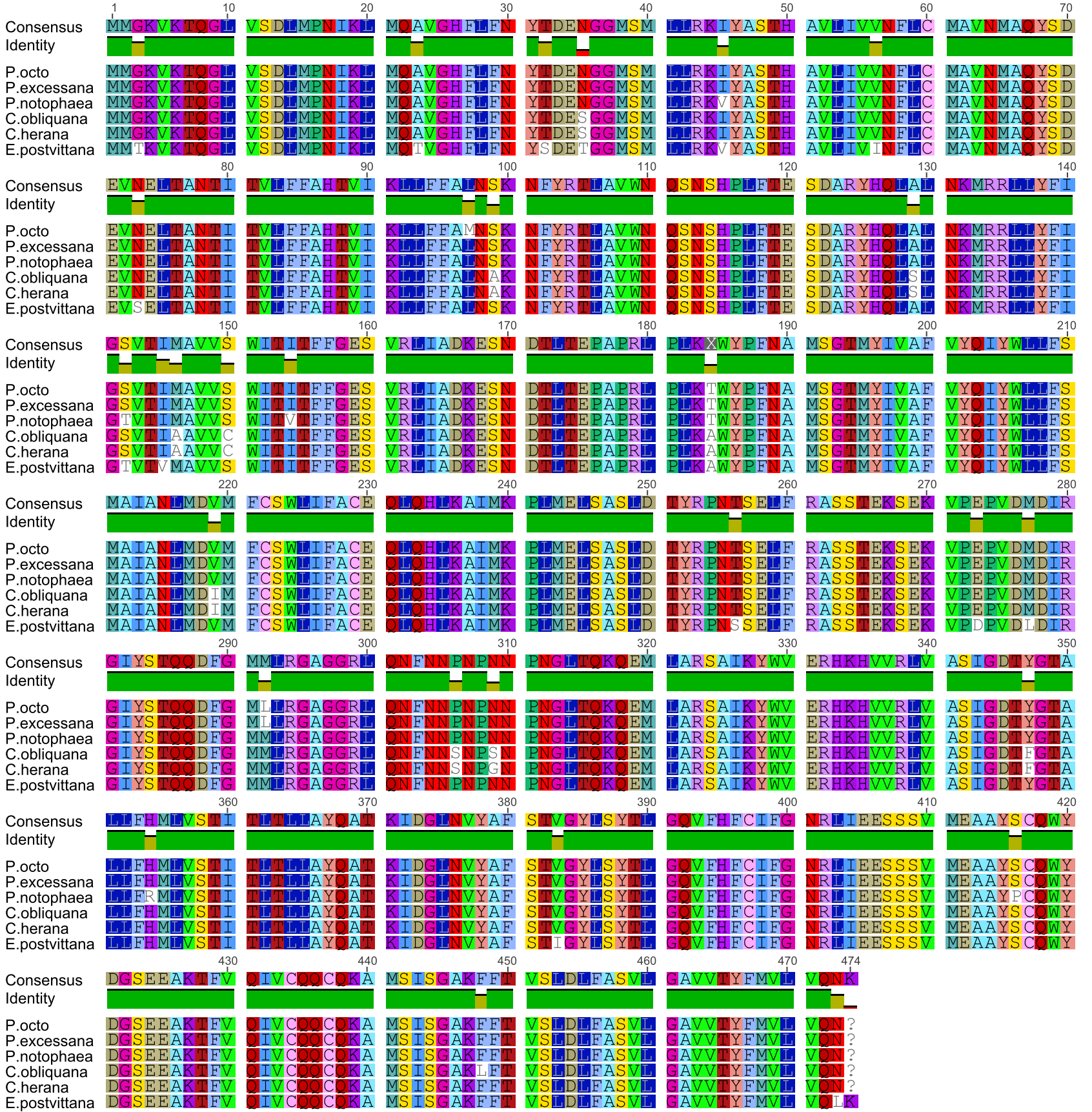

Supplement: Figure S2 — Amino acid alignment of OR2 from Planotortrix octo, P. excessana, P. notophaea Ctenopseustis obliquana, C. herana and Epiphyas postvittana. (PDF) [file pone.0038391.s002.pdf]

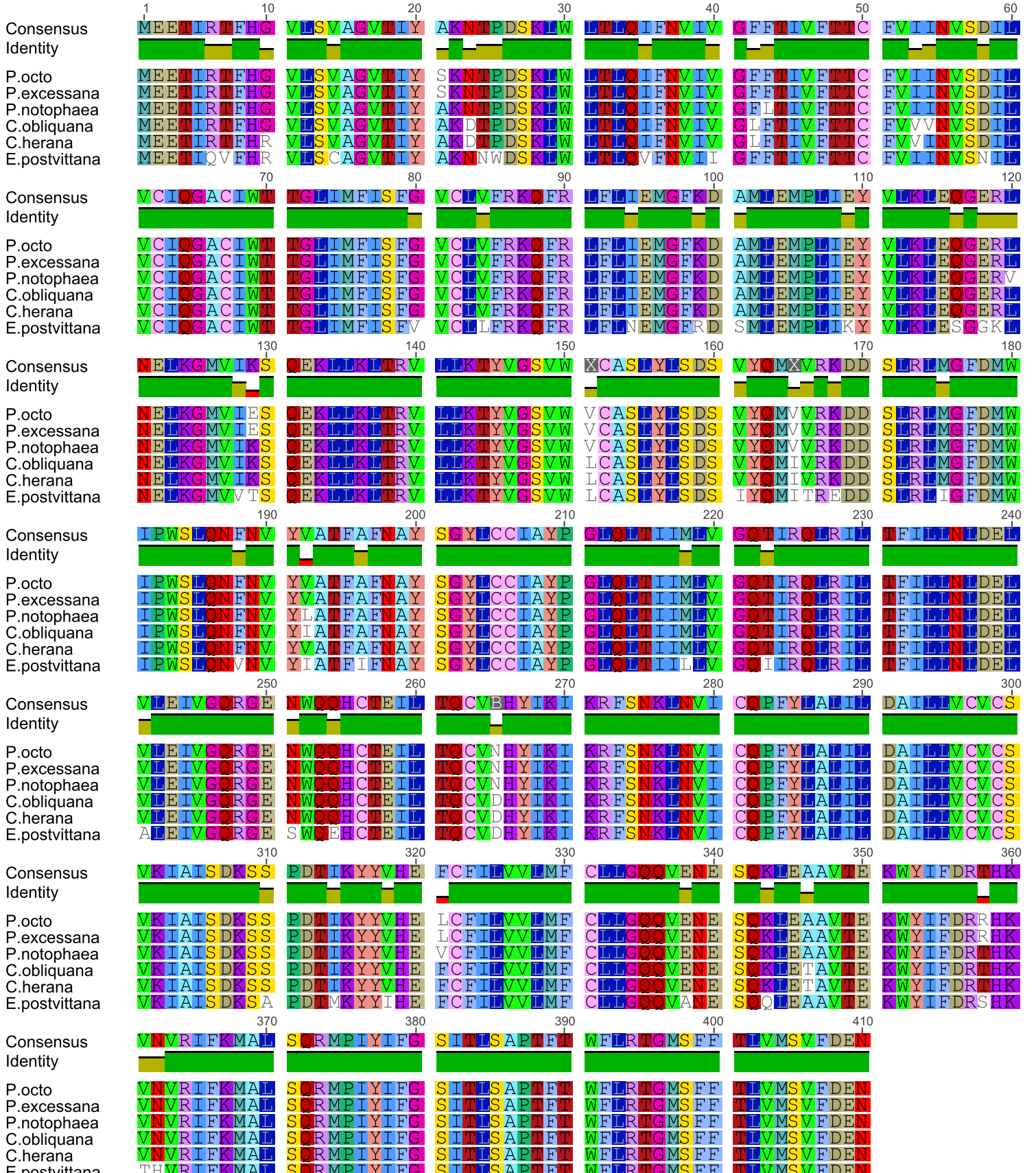

Supplement: Figure S3 — Amino acid alignment of OR3 from Planotortrix octo, P. excessana, P. notophaea Ctenopseustis obliquana, C. herana and Epiphyas postvittana. (PDF) [file pone.0038391.s003.pdf]
