# Supplementary material for: Sequence Comparisons of Odorant Receptors among Tortricid Moths Reveal Different Rates of Molecular Evolution among Family Members
Source: PLoS One. 2012 Jun 11;7(6):e38391. doi: 10.1371/journal.pone.0038391 (PMC3372514; doi:10.1371/journal.pone.0038391)
Supplement: Figure S4 — Non-synonymous and synonymous rates for all branches of OR1, OR2 and OR3 trees of Ctenopseustis obliquana , C. herana , Planotortrix octo , P. excessana and P. notophaea , with Epiphyas postvittana as an outgroup, plotted onto a consensus tree. Rates were calculated based on the M3 model in PAML, which has three categories of site with ω free to vary for each site category. (PDF) [file pone.0038391.s004.pdf]

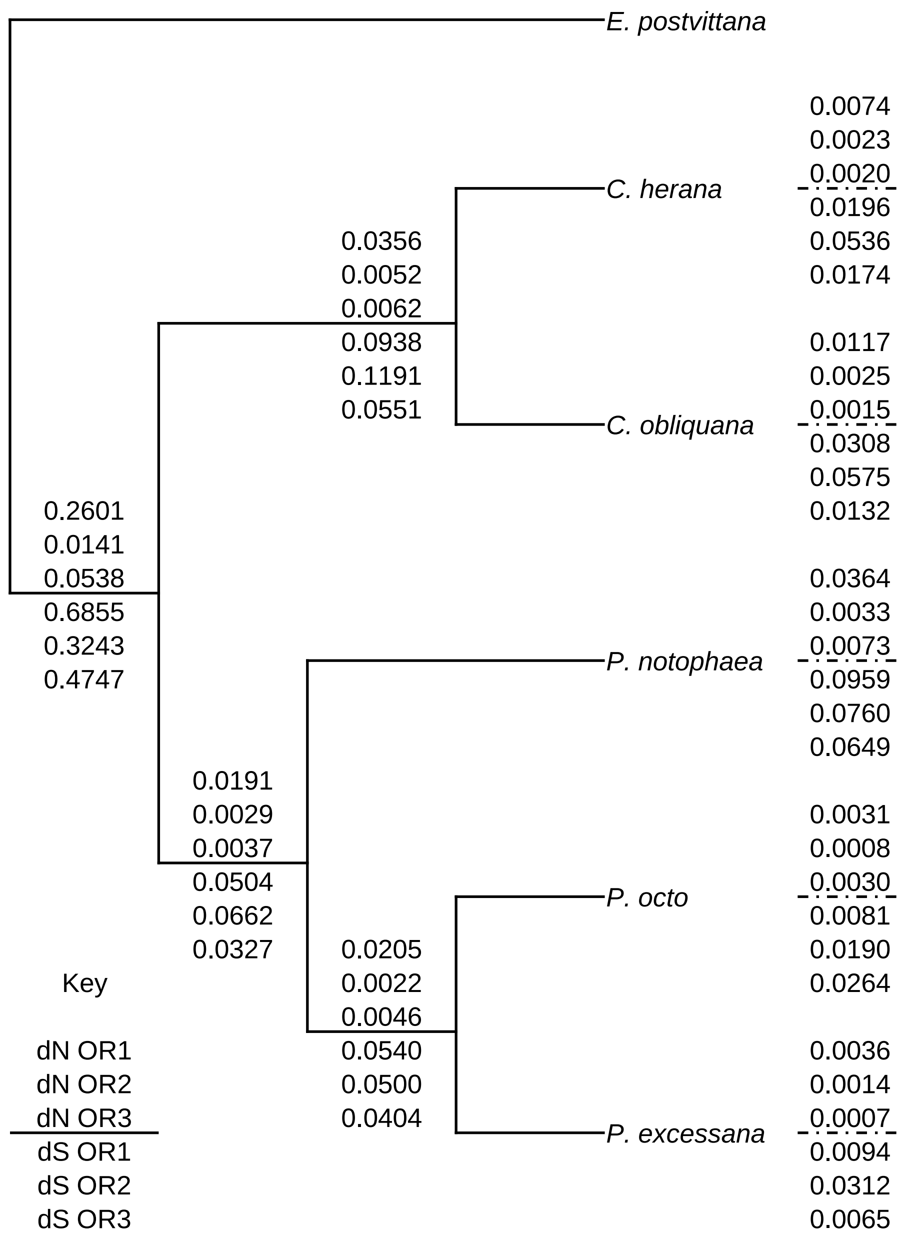

Non-synonymous and synonymous rates for all branches of OR1 , OR2 (Orco) and OR3 trees of *Ctenopseustis obliquana*, *C. herana*, *Planotortrix octo*, *P. excessana* and *P. notophaea*, with *Epiphyas postvittana* as an outgroup, plotted onto a consensus tree. Rates were calculated based on the M3 model in PAML, which has three categories of site with ω free to vary for each site category.
