# Supplementary material for: Sequence Comparisons of Odorant Receptors among Tortricid Moths Reveal Different Rates of Molecular Evolution among Family Members
Source: PLoS One. 2012 Jun 11;7(6):e38391. doi: 10.1371/journal.pone.0038391 (PMC3372514; doi:10.1371/journal.pone.0038391)
Supplement: Figure S5 — Ratio of OR1, OR2 and OR3 to α-tubulin and EF-1α from genomic DNA. (PDF) [file pone.0038391.s005.pdf]

A

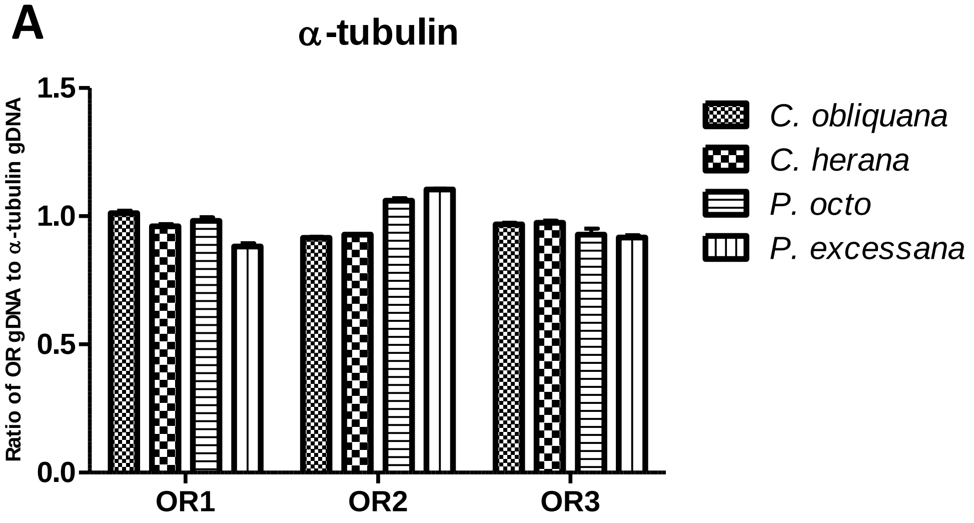

B

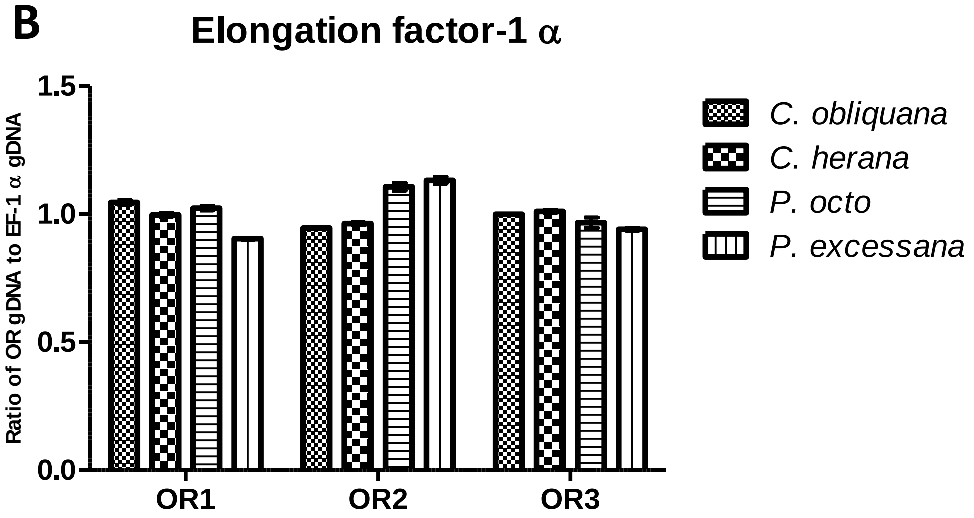

Figures: **Ratio of OR 1, OR 2 and OR 3 gDNA to α-tubulin (A) and EF-1 α (B) gDNA**. Quantitative real-time PCRs were performed on the LightCycler480 Real-Time Instrument (Roche Diagnostics, Basel, Switzerland), in a final reaction volume of 10 µL, with 20 ng of genomic DNA, 5 µL 2X SYBR Green Mix (Bio-Rad), and 2 µM of each primer. PCR cycling conditions were set to 2 min at 95°C followed by 45 cycles of 15 s at 95°C, 30 s at 60°C and 30 s at 72 °C. A final dissociation curve analysis was added (15 s at 95°C, 15 s at 60°C, and a gradual heating to 95°C at 0.01°C/s) to confirm the presence of a single amplicon [1]. Cp-values for three biological replicates (with three technical replicates each) per gene and housekeeper were collected and averaged. Averaged Cp-values of OR genes are displayed as the ratio to averaged Cp-values of α-tubulin and EF-1 α, respectively.

1. Albre J. et al. (2012) Sex pheromone evolution is associated with differential regulation of the same desaturase gene in two genera of leafroller moths. PLoS Genetics **8**: e1002489.
